# 

**Published:** 1894-04-21

**Authors:** 


PRICE TWOPENCE.

No. 395, Vol. XVI.—April 21st, 1894.

[Registered at the General Post Office as a Newspaper for transmission in the United Kingdom and Abroad.]

WITH EXTRA NURSING SUPPLEMENT.
Per Annum, 8s. 6d., post free.
Monthly Parts, 6d.; post free, 8d.
Ditto per Annum, 8s., post free.

### THE HOSPITAL

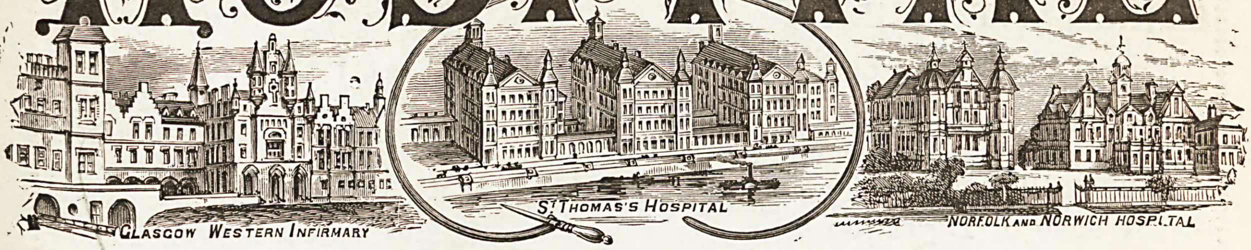

No. 395. Vol. XVI. WITH EXTRA NURSING SUPPLEMENT. SATURDAY, APRIL 21ST, 1894.

